# Construction and Annotation of a High Density SNP Linkage Map of the Atlantic Salmon (*Salmo salar*) Genome

**DOI:** 10.1534/g3.116.029009

**Published:** 2016-05-17

**Authors:** Hsin Y. Tsai, Diego Robledo, Natalie R. Lowe, Michael Bekaert, John B. Taggart, James E. Bron, Ross D. Houston

**Affiliations:** * The Roslin Institute and Royal (Dick) School of Veterinary Studies, University of Edinburgh, Midlothian, EH25 9RG, UK; † Departamento de Genética, Facultad de Biología, Universidad de Santiago de Compostela, 15782, Spain; ‡ Institute of Aquaculture, School of Natural Sciences, University of Stirling, FK9 4LA, UK

**Keywords:** *Salmo salar*, linkage map, SNP array, recombination, RNA-Seq

## Abstract

High density linkage maps are useful tools for fine-scale mapping of quantitative trait loci, and characterization of the recombination landscape of a species’ genome. Genomic resources for Atlantic salmon (*Salmo salar*) include a well-assembled reference genome, and high density single nucleotide polymorphism (SNP) arrays. Our aim was to create a high density linkage map, and to align it with the reference genome assembly. Over 96,000 SNPs were mapped and ordered on the 29 salmon linkage groups using a pedigreed population comprising 622 fish from 60 nuclear families, all genotyped with the ‘ssalar01’ high density SNP array. The number of SNPs per group showed a high positive correlation with physical chromosome length (*r* = 0.95). While the order of markers on the genetic and physical maps was generally consistent, areas of discrepancy were identified. Approximately 6.5% of the previously unmapped reference genome sequence was assigned to chromosomes using the linkage map. Male recombination rate was lower than females across the vast majority of the genome, but with a notable peak in subtelomeric regions. Finally, using RNA-Seq data to annotate the reference genome, the mapped SNPs were categorized according to their predicted function, including annotation of ∼2500 putative nonsynonymous variants. The highest density SNP linkage map for any salmonid species has been created, annotated, and integrated with the Atlantic salmon reference genome assembly. This map highlights the marked heterochiasmy of salmon, and provides a useful resource for salmonid genetics and genomics research.

Linkage maps are valuable tools for the investigation of the genetic basis of complex traits in farmed animal species. For several decades, linkage maps have enabled the mapping of quantitative trait loci (QTL), and formed the basis of attempts at positional cloning of these QTL in both terrestrial (Goddard and Hayes 2009) and aquatic farmed species (Danzmann and Gharbi 2001). High throughput sequencing technologies have now expedited the discovery of millions of single nucleotide polymorphism (SNP) markers (Liu 2010). These SNPs form the basis of modern, high-resolution genetics studies, and underpin genomic selection for faster genetic improvement in terrestrial livestock, and, laterally, aquaculture breeding programs (Meuwissen *et al.* 2001; Goddard *et al.* 2010; Sonesson 2010; Yáñez *et al.* 2014, 2015). Scoring of genome-wide SNPs in large populations is achieved either through genotyping by sequencing (Davey *et al.* 2011), or by creation and application of SNP arrays (*e.g.*, Houston *et al.* 2014a; Yáñez *et al.* 2016). High density linkage maps based on these SNP datasets can aid in high resolution mapping of loci underpinning complex traits in farmed animals (*e.g.*, Shi *et al.* 2014; Wang *et al.* 2015), improvements in assembly of reference sequences (Fierst 2015), and knowledge of the recombination landscape of the genome (*e.g.*, Groenen *et al.* 2009; Tortereau *et al.* 2012).

Reference genome assemblies are now available for several aquaculture species, including Atlantic salmon (Davidson *et al.* 2010; Lien *et al.* 2016). Once anchored and annotated, these genome assemblies provide invaluable physical maps of the genome. Due to a recent whole genome duplication, and the relatively high frequency of long and diverse repeat elements (de Boer *et al.* 2007; Davidson *et al.* 2010; Lien *et al.* 2016), assembly of the Atlantic salmon genome has been challenging, with ∼22% of the current assembly (NCBI GCA_000233375.4) yet to be assigned to chromosome. Salmonid species exhibit marked heterochiasmy, with males showing very low recombination rates across much of the genome, but with much higher recombination rates thought to occur in telomeric regions (*e.g.*, Sakamoto *et al.* 2000; Lien *et al.* 2011; Miller *et al.* 2011; Brieuc *et al.* 2014; Gonen *et al.* 2014). This phenomenon may be related to the pairing and recombination between homeologous regions of the genome, particularly in males (Wright *et al.* 1983; Allendorf and Thorgaard 1984; Allendorf *et al.* 2015). Several high density SNP arrays exist for Atlantic salmon (Houston *et al.* 2014a; Yáñez *et al.* 2016), and integrated linkage maps based on those arrays would facilitate detailed interrogation of the unusual recombination landscape. Further, while the high density SNP arrays have been applied for genome-wide association study (GWAS) and genomic prediction (Ødegård *et al.* 2014; Correa *et al.* 2015; Tsai *et al.* 2015; Tsai *et al.* 2016), such studies would be enhanced by annotation of the SNPs according to their genomic position, nearby genes, and their predicted effects.

Therefore, the purposes of this study were: (i) to construct a linkage map of the SNPs contained on the publicly available high density Affymetrix Atlantic salmon SNP array ‘ssalar01’ (Houston *et al.* 2014); (ii) to align and compare the linkage map to the latest Atlantic salmon reference genome assembly (GenBank assembly accession GCA_000233375.4); (iii) to assign previously unmapped reference genome contigs and genes to chromosomes; (iv) to investigate and compare patterns of male and female recombination across the genome; and (v) to annotate the SNPs according to their position relative to putative genes, including prediction of variant effects.

## Materials and Methods

### Animals

The population used for the linkage analysis was a subset of those described in Gharbi *et al.* (2015), purchased from Landcatch Natural Selection (LNS, Ormsary, UK). The juvenile fish used in the current study were from the 2007 year group of the LNS broodstock, and were from 60 full sibling families (28 sires and 60 dams) comprising at least six progeny per family. The trial (which focused on resistance to sea lice) was performed by Marine Environmental Research Laboratory (Machrihanish, UK), and under approval of ethics review committee in the University of Stirling (UK). Full details of the trial, and the population used, have been described previously (Houston *et al.* 2014b; Gharbi *et al.* 2015; Tsai *et al.* 2015, 2016).

### SNP array genotyping

Genomic DNA from each sample was extracted (Qiagen, Crawley, UK), and genotyped for the ‘ssalar01’ Affymetrix Axiom SNP array containing ∼132,000 validated SNPs. Details of the creation and testing of the SNP array are given in Houston *et al.* (2014a). Details of the quality control filtering of the genotypes are given in Tsai *et al.* (2015). Briefly, the Plink software was used to filter the validated SNPs by removing individuals and SNPs with excessive (> 1%) Mendelian errors, and SNPs with minor allele frequency (MAF) < 0.05 in this dataset. In total, 111,908 SNPs were retained for 622 fish (534 offspring, 28 sires, and 60 dams). Details of all the SNP markers are available at dbSNP ​(Sherry *et al.* 2001) (NCBI ss# 947429275–947844429.)

### Linkage analysis

Lep-Map2 (Rastas *et al.* 2016) was used to construct the linkage maps. The ‘Filtering’ function was applied to the initial input dataset, with ‘MAFLimit’ set at 0.05 (consistent with filtering described above), and ‘dataTolerance’ set at 0.001 to remove markers exhibiting significant segregation distortion. The ‘SeparateChromosomes’ function was applied to cluster markers into linkage groups, with the LOD threshold of 36 applied (chosen because this is the level at which 29 groups were formed, consistent with the expected karyotype of European Atlantic salmon). The function ‘JoinSingles’ was applied to assign additional single SNPs to existing linkage groups. Subsequently, the function ‘OrderMarkers’ was applied to estimate the marker order within each linkage group. Using parallelized computing, this step was repeated several times to assess consistency of marker order between replicates. Sex-specific linkage maps were generated because of the known difference in recombination rate between male and female Atlantic salmon (Gilbey *et al.* 2004; Moen *et al.* 2004; Lien *et al.* 2011; Gonen *et al.* 2014). To compare the genetic and physical maps, the flanking sequence for each SNP locus (35 bp either side) was aligned with the Atlantic salmon reference genome assembly (GenBank assembly GCA_000233375.4) (Davidson *et al.* 2010), and only complete and exact matches to the reference genome (e-value = 3 × 10^−29^) were retained. In cases where the SNP flanking sequence aligned exactly with > 1 genomic region, the alignment corresponding to the chromosome that was consistent with the linkage mapping of the SNP was retained.

### RNA sequencing

Atlantic salmon fry samples from two different families from the Scottish breeding nucleus of Landcatch Natural Selection Ltd were selected for RNA sequencing, corresponding to families ‘B’ and ‘S’ in Houston *et al.* (2010). Full details of the library preparation and sequencing are given in Houston *et al.* (2014a) (although for the current study, only two of the three families previously sequenced were used for assembling the transcriptome. This was because the third family, ‘C’, had large variation in sequence coverage between samples). Briefly, a total of 48 individual fry were homogenized in 5 ml TRI Reagent (Sigma, St. Louis, MO) using a Polytron mechanical homogenizer (Kinemetica, Switzerland). The RNA was isolated from 1 ml of the homogenate, using 0.5 vol of RNA precipitation solution (1.2 mol/l sodium chloride; 0.8 mol/l sodium citrate sesquihydrate), and 0.5 vol isopropanol. Following resuspension in nuclease-free water, the RNA was purified using the RNeasy Mini kit (Qiagen, UK). The RNA integrity numbers from the Bioanalyzer 2100 (Agilent, Santa Clara, CA) were all over 9.9. Thereafter, the Illumina Truseq RNA Sample Preparation kit v1 protocol was followed directly, using 4 μg of RNA per sample as starting material. Libraries were checked for quality, and quantified using the Bioanalyzer 2100 (Agilent), before being sequenced in barcoded pools of 12 individual fish on the Illumina Hisequation 2000 instrument (100 base paired-end sequencing, v3 chemistry); all sequence data were deposited in the European Nucleotide Archive under accession number ERP003968.

### Transcriptome assembly

The quality of the sequencing output was assessed using FastQC (http://www.bioinformatics.babraham.ac.uk/projects/fastqc/; version 0.11.2). Quality filtering and removal of residual adaptor sequences was conducted on read pairs using Trimmomatic v.0.32 (Bolger *et al.* 2014). Specifically, residual Illumina specific adaptors were clipped from the reads, leading and trailing bases with a Phred score less than 15 were removed, and the read trimmed if a sliding window average Phred score over four bases was less than 20. Only paired-end reads where both sequences had a length greater than 36 bases postfiltering were retained. The most recent salmon genome assembly (ICSASG_v2, NCBI assembly GCA_000233375.4) was used as a reference for read mapping. Filtered reads were mapped to the genome using Tophat2 v. 2.0.12 (Kim *et al.* 2013), which leverages the short read aligner Bowtie2 v.2.2.3 (Langmead and Salzberg 2012), allowing a maximum of two mismatches. Using Cuffdiff v.2.2.1 (Trapnell *et al.* 2012), the aligned reads were merged into a transcriptome assembly. The transcriptome was annotated against NCBI nonredundant protein and nucleic acid databases using local Blast v.2.3.0+ (Altschul *et al.* 1997) with a cut-off e-value of 10^−5^. The completeness of the salmon transcriptome was evaluated using Blast searches with a cut-off e-value of 10^−25^ against a set of 248 core eukaryotic genes (Parra *et al.* 2007).

### SNP annotation

For every gene, the most highly expressed transcript variant was selected to identify candidate coding regions using Transdecoder v.2.0.1 (http://transdecoder.sourceforge.net/). Open reading frames (ORF) were predicted for every transcript, requiring a minimum of 100 amino acids (to reduce the number of potential false positives). All the predicted proteins were aligned against the manually curated UniRef90 database using local Blast v.2.3.0+ (Altschul *et al.* 1997) with a cut-off e-value of 10^−5^, discarding ORFs without positive matches. Finally, the longest ORF was selected as the canonical protein for each transcript. The final set of coding regions was used to build a genome annotation file which was used to predict the functional significance of all the SNPs on the ‘ssalar01’ SNP array using SnpEff v.4.2 (Cingolani *et al.* 2012).

### Data availability

The authors state that all data necessary for confirming the conclusions presented in the article are represented fully within the article.

## Results and Discussion

### Linkage map construction

**Figure 1:**
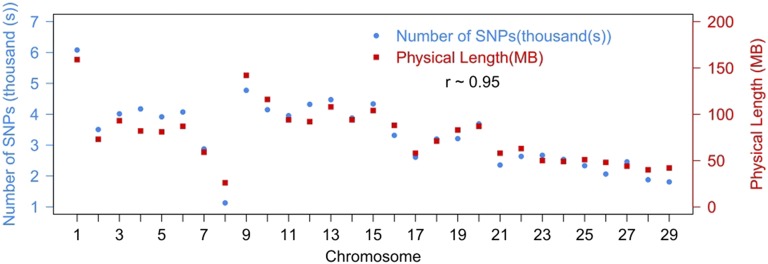
Comparison of the number of SNPs in corresponding chromosomes and physical length retrieving from recent reference assembly (GenBank assembly reference GCA_000233375.4, Davidson *et al.* 2010). The correlation was approximately 0.95.

A pedigreed population of 622 individual Atlantic salmon (534 offspring, 28 sires, and 60 dams) were successfully genotyped using the high density Affymetrix SNP array ‘ssalar01’ (Houston *et al.* 2014a). SNPs were assigned to putative linkage groups, and then ordered on each linkage group using Lep-Map2 (Rastas *et al.* 2016). A total of 111,908 SNPs was retained following QC filtering, of which 96,396 (86%) were assigned and ordered on the 29 linkage groups (which correspond to the karyotype of European Atlantic salmon). The number of SNPs per chromosome varied from 1128 to 6080, and was positively correlated with the number of SNPs per chromosome in previously published Atlantic salmon SNP linkage maps of Lien *et al.* (2011) (*r* = 0.94), and Gonen *et al.* (2014) (*r* = 0.87). The flanking sequences of the SNPs on the linkage map were aligned to the salmon reference genome assembly (GCA_000233375.4) to determine their putative physical position (Supplemental Material, File S1). There was a high positive correlation between the genetic map position and the reference sequence position of the SNPs (Table 1), and the number of SNPs per chromosome was dependent on chromosome sequence length (Figure 1). SNP density for the successfully genotyped and mapped markers from the ‘ssalar1’ array is relatively constant across the genome, with an average of 1 SNP per ∼23 kb in the assembled chromosomes, and 1 SNP per 0.05 cM (male) and 0.07 cM (female) in the full linkage map.

**Table 1:** The characteristics of the physical and genetic maps of the 29 Atlantic salmon (pairs of) chromosomes (GenBank reference GCA_000233375.4; Davidson *et al.* 2010)

				Male		Female	
Chr.	SNPs	Physical Length (MB)*^a^*	Physical Length of Unassigned Contigs (MB)*^a^*	Max (cM)	Correlation*^b^*	Max (cM)	Correlation*^b^*
1	6080	159	1.6	428.8	0.97	551.3	0.98
2	3506	73	3.1	173.5	0.80	404.4	0.85
3	4013	93	2.2	332.2	0.84	467.7	0.96
4	4173	82	1.1	156.6	0.82	183.6	0.95
5	3916	81	1.9	274.4	0.91	529.9	0.93
6	4073	87	2.3	264.2	0.88	689.1	0.89
7	2875	59	1.2	183.7	0.85	249.0	0.97
8	1128	26	0.6	181.6	0.87	326.4	0.97
9	4774	142	1.7	278.8	0.77	392.2	0.81
10	4146	116	0.9	82.8	0.79	166.8	0.97
11	3953	94	2.8	166.2	0.79	291.0	0.81
12	4321	92	2.6	95.7	0.80	239.5	0.80
13	4472	108	1.3	178.0	0.62	213.8	0.91
14	3878	94	1.4	96.4	0.73	123.5	0.92
15	4335	104	1.9	77.3	0.64	136.9	0.91
16	3316	88	2.3	141.9	0.80	137.7	0.90
17	2607	58	2.0	171.2	0.90	307.2	0.96
18	3196	71	1.4	91.7	0.85	105.9	0.92
19	3210	83	1.5	74.5	0.76	103.2	0.90
20	3687	87	1.5	96.5	0.82	112.5	0.93
21	2355	58	0.7	93.2	0.80	159.1	0.84
22	2634	63	0.4	73.6	0.74	78.0	0.88
23	2670	50	0.6	77.5	0.65	84.4	0.96
24	2538	49	0.3	379.0	0.91	458.2	0.97
25	2332	51	0.7	147.0	0.92	175.3	0.96
26	2063	48	2.2	166.2	0.92	161.8	0.95
27	2458	44	0.4	73.3	0.72	72.6	0.91
28	1878	40	0.7	143.1	0.94	156.0	0.99
29	1809	42	0.6	70.2	0.73	76.4	0.88
Total	96,396	2242	41.9	4769.0	—	7153.2	—
Avg	3324	77	1.4	164.5	0.81	246.7	0.92

a The physical length is taken from the latest Atlantic salmon genome assembly [GenBank reference GCA_000233375.4 (Davidson *et al.* 2010)], and ‘unassigned contigs’ are those that were unplaced on the reference assembly but mapped to the chromosome in the linkage map.

b The correlation between the genetic distance of SNPs (cM) on the linkage map and the physical distance (bp) according to the reference genome assembly.

The most recent Atlantic salmon reference genome assembly (GCA_000233375.4) contains 2240 MB of sequence contigs anchored to chromosomes (78% of total assembly), and 647 MB of contigs that are not yet assigned to chromosome (22% of total assembly). Linkage mapping using high density SNP arrays was applied to orientate reference genome contigs and scaffolds, and to identify putative misassemblies in the recently published salmon genome paper (Lien *et al.* 2016). However, those linkage maps are unpublished. In the current study, a total of 4581 previously unassigned contigs comprising 41.9 MB of sequence was tentatively mapped to the 29 salmon chromosomes (Table 1 and File S2). While additional experiments would be required to confirm the correct position of these genome contigs, this linkage map has enabled an additional ∼1% of the entire reference genome assembly to be tentatively mapped to chromosomes, corresponding to ∼6.5% of the previously unassigned genome assembly. These contigs were spread across all 29 chromosome pairs (Table 1, and details given in File S1). Novel potentially misassembled regions were also identified in the reference sequence via regions of discordance between the linkage and physical maps, an example of which is between ∼11.5 MB and 11.8 MB on Chromosome 26 (File S3).

**Figure 2:**
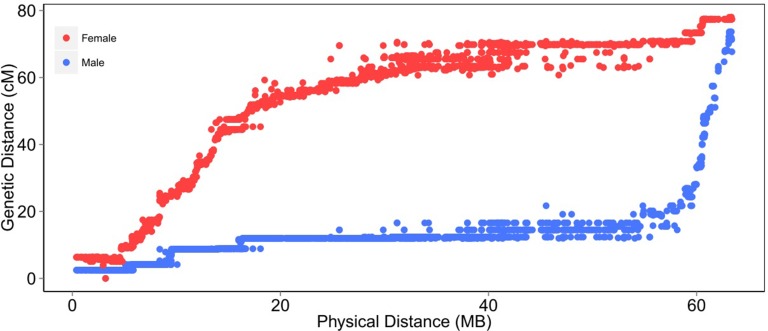
A comparison between genetic and physical maps of a representative chromosome (Chr 22), reflecting the recombination pattern difference between males and females. Details of genetic distance and physical distance for all mapped loci are given in File S1.

**Figure 3:**
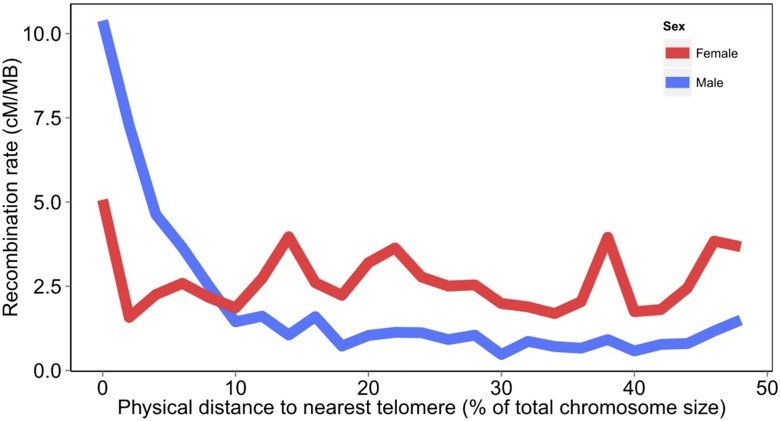
A comparison of male and female recombination level (cM/Mb) graphed according to physical distance from the nearest chromosome end (expressed as a percentage of total chromosome size in megabases).

There were substantial differences in the patterns of recombination between the sexes. The female linkage map covered 7153 cM (ranging from 72.6 to 689.0 cM per chromosome), whereas the male linkage map covered 4769 cM (ranging from 70.2 to 428.8 cM per chromosome) (Table 1). Overall, the female map was ∼1.5 × longer than the male map, consistent with previous Atlantic salmon SNP linkage maps (Lien *et al.* 2011; Gonen *et al.* 2014). The pattern of recombination across the genome was notably different between the sexes, with female recombination rates being higher across much of the genome, except for some subtelomeric regions where male recombination was substantially higher (*e.g.*, Figure 2). This phenomenon has been observed in several previous salmonid linkage maps (Sakamoto *et al.* 2000; Lien *et al.* 2011; Miller *et al.* 2011; Brieuc *et al.* 2014; Gonen *et al.* 2014), but the availability of the reference genome enables a more detailed investigation. Therefore, linkage and physical maps were aligned, and a proxy of recombination rate (number of centimorgans per megabase) was estimated at regular intervals on each chromosome, with each interval corresponding to 2% of the total chromosome’s physical length. The average recombination rate for each corresponding interval on the 29 chromosomes was calculated and graphed against the distance from the nearest telomere (Figure 3). The results highlight the phenomenon of markedly high male recombination in some subtelomeric regions, on average ∼10 × higher than regions of the genome nearer the middle of the chromosome (Figure 3).

### Transcriptome assembly and annotation

To annotate the mapped SNPs and predict their function according to their position relative to putative genes, an annotated reference transcriptome was created. RNA-seq of 48 individual salmon fry yielded 927 M raw paired-end sequence reads, of which 93% remained after trimming and filtering. Filtered reads were aligned to the most recent Atlantic salmon reference genome assembly (GCA_000233375.4; 82.2% concordant pair alignment) to generate a reference transcriptome. The alignment resolved 202,009 putative transcripts corresponding to 65,803 putative genes, consisting of 36,846 single transcript genes, and 28,957 multi-transcript genes (Table 2 and File S4). The average length of the transcripts was 4127 bp, with an N50 of 5710, an N90 of 2323, and > 90% of transcripts longer than 500 bp. The assembled transcripts were annotated using BLASTx and BLASTn searches against the NCBI nonredundant protein and nucleic acid databases, respectively. Of the 65,803 total putative genes, 58,416 (88.8%) showed significant similarity to known proteins, while an additional 2732 (4.2%) showed significant similarity to nucleotide entries in the NCBI nonredundant nucleotide database (File S5). The proportion of unannotated genes was higher for the shorter transcript sequences (File S6), but all transcripts were retained (since a relevant minimum size threshold was not apparent). The completeness of the transcriptome was evaluated against a set of 248 core eukaryotic genes described in Parra *et al.* (2007); 247 of these genes were found in our transcriptome (BLASTn e-value < E10^−25^), 222 of which had at least 90% coverage, and 153 of which were fully covered. A total of 53,950 identified genes was located within chromosomes on the Atlantic salmon genome assembly, while the remaining 11,853 were aligned to unassigned contigs. Of these 11,853 genes, 1647 (13.9%) were located in contigs assigned to chromosomes using the linkage map of the current study (Table 1 and File S7).

**Table 2:** Summary statistics for the Atlantic salmon RNA-seq transcriptome assembly

Transcriptome assembly details	Number
Transcripts	202,009
Genes	65,803
Single transcript genes	36,846
Multi-transcript genes	28,957
Genes in assembled chromosomes	53,950
Genes in unassigned contigs	11,853
Average transcript length	4127
N50	5710
N90	2323
Transcripts > 500 bp	195,224
Genes annotated using protein database	58,416
Genes annotated using DNA database	2732

### SNP annotation

The RNA-seq based transcriptome described above was used to predict ORFs and protein sequences in order to annotate the SNPs present on the ‘ssalar01’ array (Table 3 and File S8). A total of 106,424 SNPs (95%) matched a single genome location, while 2857 SNPs matched two different genomic positions, related in part to the salmonid specific genomic duplication. An additional 880 SNPs mapped to three or more genome locations, indicative of repetitive elements or protein domains. It should be noted that filtering of SNPs during the design process for the array would have removed the majority of SNPs mapping to two or more locations (Houston *et al.* 2014a). The tentative annotation of all SNPs is given (File S6), but only those mapping to unique genomic regions are described below. Of these 106,424 unique SNPs, 48,842 (45.9%) were located in putative genes, with the remainder mapping to intergenic regions. Of the genic SNPs, the majority were in putative intronic regions (34,534; 70.7%), although 483 of these were associated with splicing regions, and therefore have a higher likelihood of being functionally relevant. The remaining genic SNPs were mapped to putative untranslated regions (UTRs; 8091), with a larger amount of SNPs in the 3′-UTR as expected (6224 *vs.* 1867 in the 5′-UTR); and to putative exons (5856). A total of 2465 putative nonsynonymous SNPs was identified, in addition to 39 SNPs predicted to cause gain/loss of start/stop codons, which have a high likelihood of functional consequences (File S8). As an example, a premature stop codon was found in phospholipase D, an enzyme which produces the signal molecule phosphatidic acid, which is also a precursor for the biosynthesis of many other lipids (McDermott *et al.* 2004). The distribution of the SNP functional categories across the 29 chromosome pairs is given in Table 4. It is important to note that these predicted SNP effects will contain a proportion of false positives due to inevitable errors in the predicted structure of the genes. Nonetheless, their annotation combined with their linkage and physical mapping provides a valuable resource for users of the high density ‘ssalar01’ array in particular, and for salmonid genomics researchers in general.

**Table 3:** Predicted numbers, location and effect of the mapped SNPs according to their position on the annotated reference genome

Summary of annotated SNPs					
Intergenic	57,582				
Genic	48,842	UTR	8091	5′	1867
				3′	6224
		Intron	34,534	Splice region	483
				Nonsplice region	34,051
		Exon	5856	Synonymous	3352
				Nonsynonymous	2465
				Gain or loss of start/stop codon	39

**Table 4:** Number of predicted genes and functional categories of SNPs split according to chromosome

Genes and SNPs per Chromosome					
Chromosome	Genes	Exonic SNPs	Intronic SNPs	UTR SNPs	Intergenic SNPs
1	3507	181	877	206	4717
2	2711	222	1116	284	1630
3	2741	225	1209	312	2026
4	2255	246	1301	309	2066
5	2286	220	1184	299	2030
6	2441	217	1286	312	2006
7	1526	152	928	192	1455
8	875	44	335	67	525
9	3062	244	1415	374	2563
10	2568	217	1341	300	2140
11	2308	162	1168	249	2207
12	2672	268	1398	349	2088
13	2524	276	1516	328	2181
14	2343	236	1154	314	2034
15	2400	271	1415	294	2138
16	2205	193	1003	253	1721
17	1770	144	744	206	1307
18	1767	142	1041	205	1654
19	1694	125	1013	203	1743
20	2072	211	1093	257	1830
21	1056	129	700	160	1252
22	1398	153	811	189	1416
23	1138	142	863	192	1400
24	1040	146	860	187	1238
25	1032	113	585	133	1431
26	1372	102	606	128	1082
27	1096	129	828	195	1221
28	912	92	593	147	992
29	821	88	598	120	937
Total	55,592	5090	28,981	6764	51,030
Avg	1917	176	999	233	1760

### Conclusion

A linkage map comprising > 96,000 SNPs from the ‘ssalar01’ array was created, annotated, and integrated with the reference genome assembly. This represents the highest density SNP linkage map for any salmonid species. Alignment of the linkage and physical maps revealed good agreement between genetic map, and the mapping allowed a further circa 1% of the salmon reference genome assembly to be tentatively assigned to chromosomes. Marked heterochiasmy was observed, with male recombination rate substantially lower than females across much of the genome, but with a notably high level in some subtelomeric regions. Finally, the mapped SNPs were annotated and categorized according to their predicted function. The map will be another useful resource for salmonid genomics research.

## Supplementary Material

Supplemental Material
