# Supplementary material for: Construction and Annotation of a High Density SNP Linkage Map of the Atlantic Salmon (Salmo salar) Genome
Source: G3 (Bethesda). 2016 May 17;6(7):2173–9. doi: 10.1534/g3.116.029009 (PMC4938670; doi:10.1534/g3.116.029009)
Supplement: Supplemental Material [file supp_6_7_2173__index.html]

Construction and Annotation of a High Density SNP Linkage Map of the Atlantic Salmon (Salmo salar) Genome — Supplemental Material 

# Construction and Annotation of a High Density SNP Linkage Map of the Atlantic Salmon (*Salmo salar*) Genome

## Supplemental Material for Tsai *et al.*, 2016

**Files in this Data Supplement:**

- File S1 - Details of the linkage map of the 29 chromosomes. (.xls, 23 MB)
- File S2 - Unassigned genome contigs. (.xls, 1 MB)
- File S3 - Potential misassembly in reference genome. (.jpg, 67 KB)
- File S4 - Reference transcriptome sequence (fasta). (.zip, 111 MB)
- File S5 - Reference transcriptome annotation. (.xlsx, 8 MB)
- File S6 - Length distribution of annotated and unannotated genes. (.tif, 368 KB)
- File S7 - Number of previously unmapped genes assigned to chromosomes using the linkage map. (.xlsx, 8 KB)
- File S8 - SNP annotation. (.xlsx, 8 MB)
