# Supplementary figures and images for: Construction and Annotation of a High Density SNP Linkage Map of the Atlantic Salmon (Salmo salar) Genome
Source: G3 (Bethesda). 2016 May 17;6(7):2173–9. doi: 10.1534/g3.116.029009 (PMC4938670; doi:10.1534/g3.116.029009)

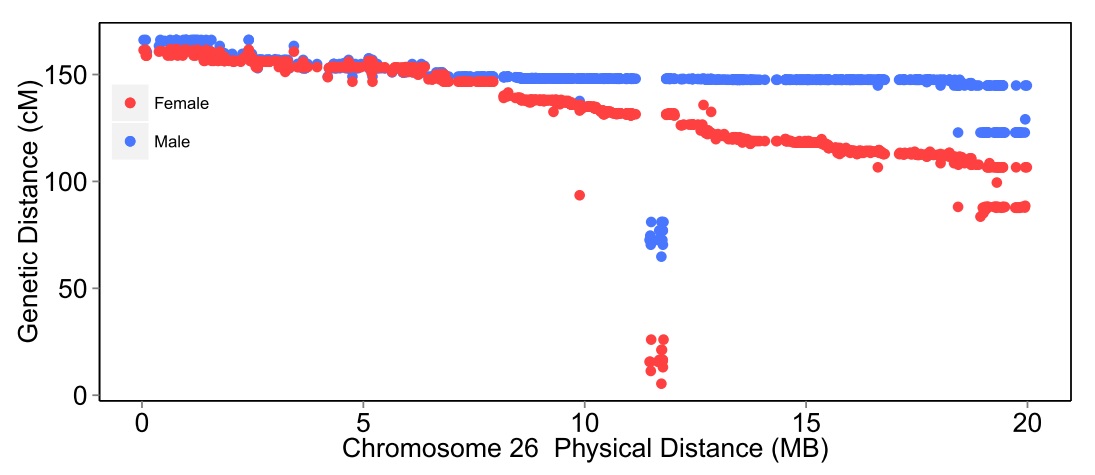

Supplement: Supplemental Material [file supp_g3.116.029009_FileS3.jpg]

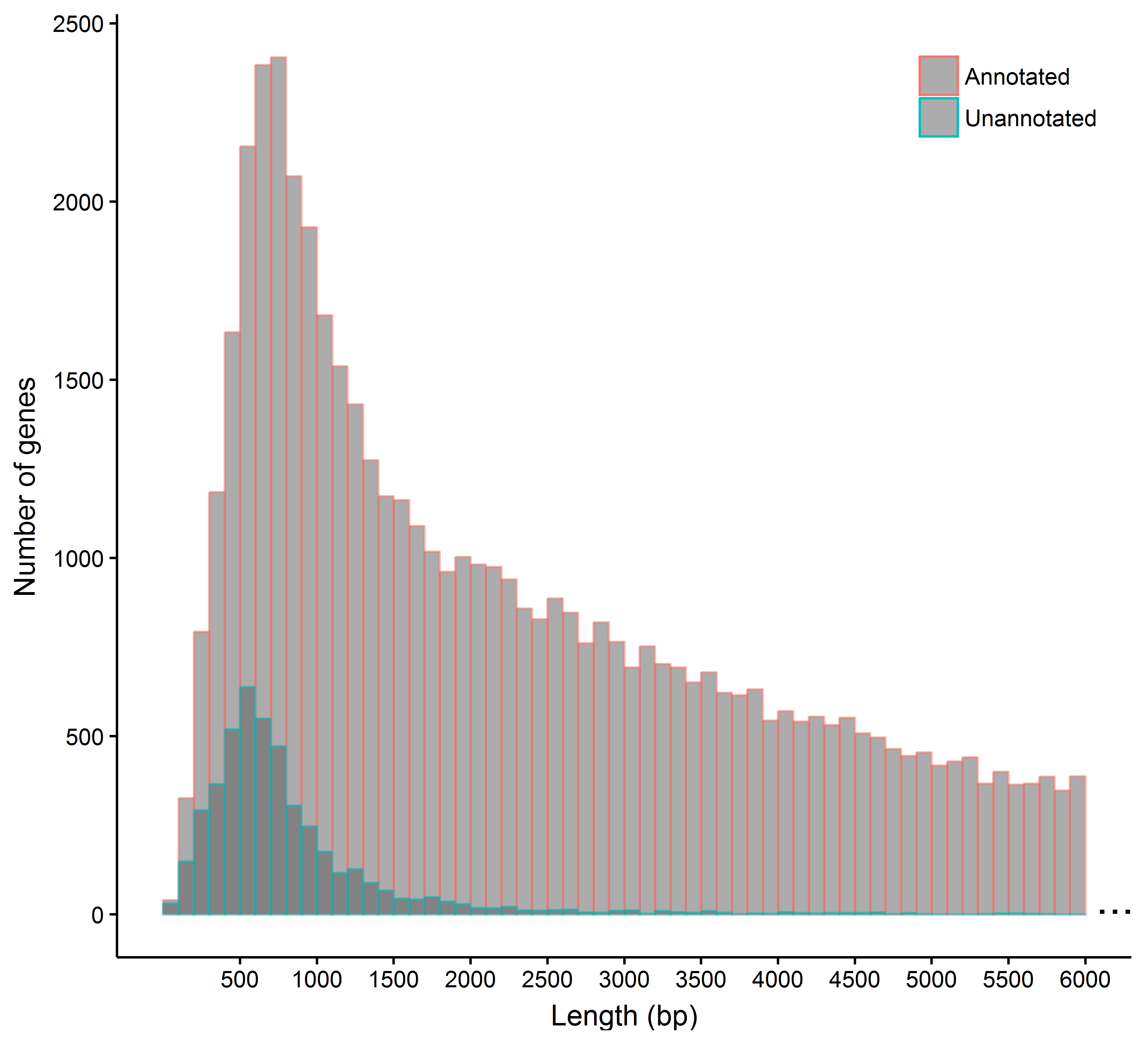

Supplement: Supplemental Material [file supp_g3.116.029009_FileS6.tif]
